# A commentary on ‘Prognosis and conditional nomogram of cervical spine fracture in patients with severe spinal cord injury: a multicenter retrospective study’

**DOI:** 10.1097/JS9.0000000000001062

**Published:** 2024-01-11

**Authors:** WenJian Wang, Desheng Kong, Xiaocong Ju, Feng Chen, Xufeng Yang

**Affiliations:** a Trauma Center, Jinan Central Hospital Affiliated to Shandong First Medical University; b Department of Oncology, Jinan Central Hospital Affiliated to Shandong First Medical University; c Department of Orthopedics, The Third Affiliated Hospital of Shandong First Medical University, Jinan, Shandong; d Department of Neurosurgery, Peking University International Hospital, Beijing, People’s Republic of China

*Dear Editor,*

Cervical traumatic spinal cord injury (CTSCI) is a severely disabling condition that frequently results in sensory and motor dysfunction below the level of injury, negatively impacting the patient’s physical and mental health and imposing a tremendous economic burden on the patient’s family and society, accounting for ~55% of all traumatic spinal cord injuries (SCI)^1^. Huang *et al*.^2^ conducted a multicenter retrospective study on patients with cervical fractures and severe SCI and developed conditional nomograms. In conclusion, conditional nomograms can be used to forecast survival probabilities for varying survival periods, aid in prognostic interpretation, and enhance collaborative decision-making techniques.

There were 450 patients included in the research between January 2012 and May 2019, after propensity score matching. In this work, the exact survival rates of survivors within predetermined periods were estimated using conditional survival (CS), and the hazard function was utilized to determine the instantaneous mortality risk of such patients. According to the study’s findings, the risk of immediate death was highest during the first 12 months following damage and can be reduced by surgical therapy, particularly early-term surgery. The 5-year CS steadily climbed from 73.3% at baseline to 88.0% after 2 years of survival. The study also sheds light on these patients’ prognoses by constructing a set of nomograms. Conditional nomograms were produced at baseline, as well as in those who lived for 6 and 12 months. The area under the receiver operating characteristic curve and the calibration curves showed that the nomograms performed well.

There were some limitations to this investigation. Firstly, the majority of the study’s patients came from northwest China, and the sample size was insufficient to adequately reflect all of Asia and China’s population^3^. Furthermore, all of the hospitals are locally associated tertiary care facilities with vast expertise in treating patients with severe SCI and cervical fractures; thus, lower-level hospitals may not fully utilize the data. Second, because this is a retrospective study, there may be bias. Finally, there is bias since the inclusion of influencing factors did not include all potential factors linked with the prognosis of patients with CTSCI. For future research to yield more compelling findings, a bigger sample size and a random selection of patients from China and even Asia would be necessary.

This study examines the danger of instantaneous mortality and CS in such patients. Furthermore, conditional nomograms were devised to account for varied periods of survivors and predict survival rates. This issue has far-reaching ramifications for clinical practice. This multicenter retrospective analysis provides preliminary evidence that surgery, especially early-term surgery, can lower the chance of immediate mortality, which was highest during the first 12 months after injury. Other studies have also verified this result^4,5^. In addition to supporting prognosis interpretation and improving cooperative decision-making strategies, conditional nomograms can be used to predict survival probability for various survival durations. This forward-thinking approach provides valuable guidance and inspiration for the continued development of this area of research. In conclusion, prognosis interpretation, collaborative decision-making, and survival probabilities for different survival periods can all be improved with the use of conditional nomograms. It can be beneficial to use past outcomes to guide clinical care plans and future trials.

## Ethical approval

Not applicable.

## Consent

Not applicable.

## Author contribution

W.W. and D.K.: study concept or design, data collection, data analysis or interpretation, and writing the paper; X.J. and F.C.: data collection; X.Y.: study concept or design, writing, and revising the paper.

## Conflicts of interest disclosure

There are no conflicts of interest.

## Research registration unique identifying number (UIN)

Not applicable.

## Guarantor

Xufeng Yang.

## Data availability statement

This manuscript is a comment. Don’t need a Data availability statement. However, all the data from the current study are publicly available.

## Provenance and peer review

Not applicable.
